# Inadvertent Subclavian Artery Cannulation: Endovascular Repair Using a Collagen Closure Device—Report of Two Cases and Review of the Literature

**DOI:** 10.1155/2012/150343

**Published:** 2012-04-04

**Authors:** Ganapathy Ananthakrishnan, Richard D. White, Rajesh Bhat, Sam Chakraverty

**Affiliations:** Department of Clinical Radiology, Ninewells Hospital and Medical School, Ninewells Avenue, Dundee DD1 9SY, UK

## Abstract

Inadvertent line insertion into the subclavian artery is an uncommon complication of subclavian venous catheterisation, and its timely recognition is vital to minimise risk of harm to the patient. We describe the radiographic, computed tomographic (CT), and angiographic findings in two patients and illustrate the subsequent endovascular management using collagen vascular closure devices.

## 1. Introduction

Subclavian vein catheterisation is a commonly performed procedure [1], with inadvertent puncture of the subclavian artery representing an uncommon but potentially fatal complication [1, 2].

We report two cases in which the subclavian artery was accidentally catheterised during attempted subclavian venous cannulation. In both cases, the catheter was removed in the interventional suite and the arterial puncture closed with an Angio-Seal vascular closure device (St. Jude Medical; St. Paul, MN). The radiographic, computed tomographic (CT), and angiographic findings are presented here. We also review the literature for management of iatrogenic subclavian arterial cannulation.

## 2. Case Presentation

**Case 1**

A 57-year-old woman presented to the accident and emergency department with severe haematemesis. The patient had undergone oesophageal dilatation one month earlier for strictures secondary to oesophagitis. The blood count revealed a drop in haemoglobin from a base level of 13.6 g/dL to 8.8 g/dL, and a decision was made to gain central venous access and commence blood transfusion.

**Figure 1:**
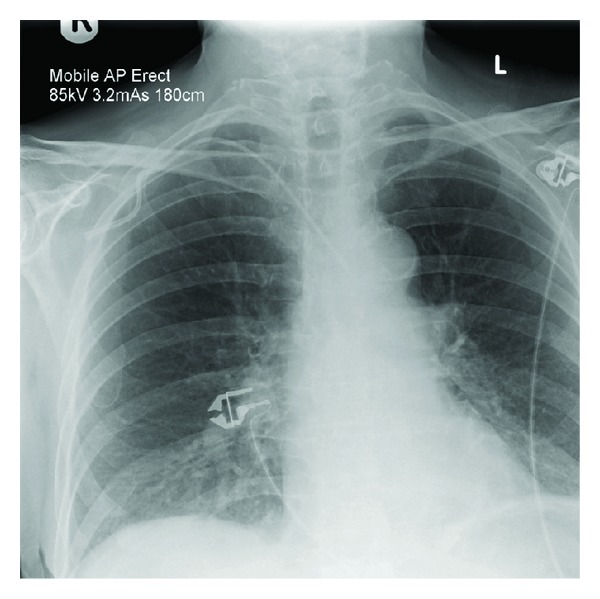
Chest radiograph showing abnormal position of the right subclavian line.

**Figure 2:**
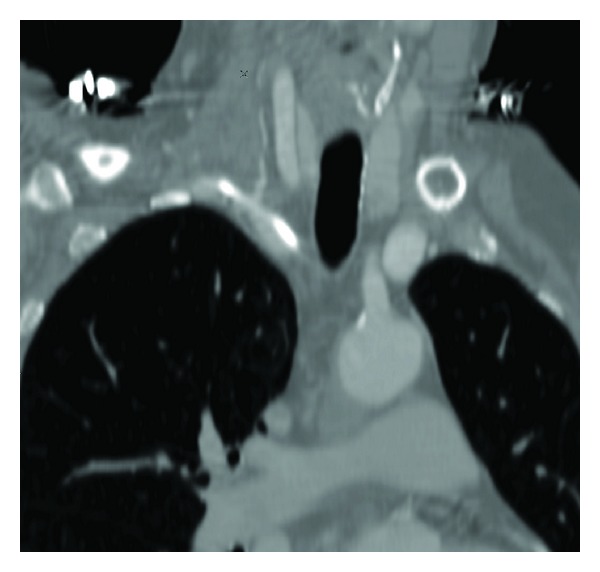
Oblique reformat of CT angiogram showing the entry site of the catheter into the artery and location of the origin of the vertebral artery.

In the High Dependency Unit (HDU), a 7 Fr catheter was introduced via a right subclavian approach. A postprocedural chest radiograph was performed (Figure 1). This showed the catheter to be projected to the left of the intended position, consistent with an inadvertent arterial insertion. CT angiography (CTA) was undertaken to confirm the misplacement (Figure 2). This demonstrated that the catheter had entered the right subclavian artery at the level of the origin of the internal mammary artery, just above the origin of the vertebral artery, with the tip lying in the aortic arch. There was no subclavian vein involvement and no mediastinal haematoma.

The following day, the patient was brought to the endovascular suite for attempted catheter removal. Under ultrasound guidance, access was gained into the right axillary artery with a 5 Fr sheath (Brite tip, Cordis) and a 4 Fr angled catheter introduced, with its tip advanced into the right brachiocephalic trunk. This was to enable angiograms to be performed at the end of the procedure and also to ensure maintenance of access across the subclavian artery, which would allow a subclavian stent to be deployed in the event of failure of the intended Angio-Seal device. We also ensured that a balloon-mounted stent was prepared and ready for deployment. Angiographic runs were also performed to help confirm that the puncture site was not excessively close to the vertebral artery origin, to avoid inadvertent occlusion with the Angio-Seal footplate.

**Figure 3:**
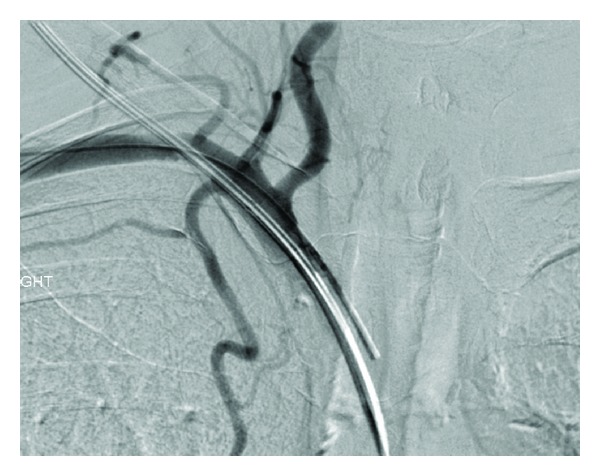
Catheter angiogram showing relation of catheter site to the right vertebral artery.

**Figure 4:**
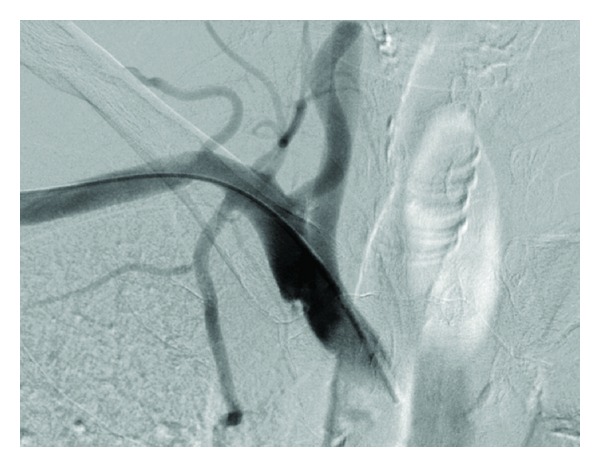
Catheter angiogram following deployment of Angio-Seal device.

A 50 cm (0.035 inch) Amplatz guidewire was inserted into the catheter. The inadvertently placed “venous” catheter was then removed and an 8 Fr Angio-Seal device deployed over the guide wire into the subclavian artery. Angiographic runs obtained from the catheter placed via the axillary artery showed no extravasation of contrast from the subclavian artery at the site of the Angio-Seal deployment (Figures 3 and 4), confirming procedural success.

**Case 2**

**Figure 5:**
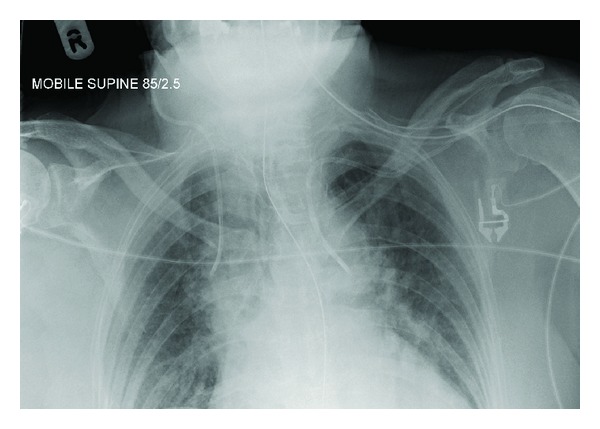
Chest radiograph showing abnormal position of the left subclavian line.

A 76-year-old female was admitted with peritonitis secondary to upper gastrointestinal visceral perforation. Following laparotomy, attempts were made to place a 4 lumen, 7 Fr left subclavian venous catheter on HDU. Blood samples obtained via the catheter were shown to be of arterial origin, and inadvertent arterial placement was confirmed on a chest radiograph, with the tip of the catheter being projected over the aortic arch (Figure 5).

**Figure 6:**
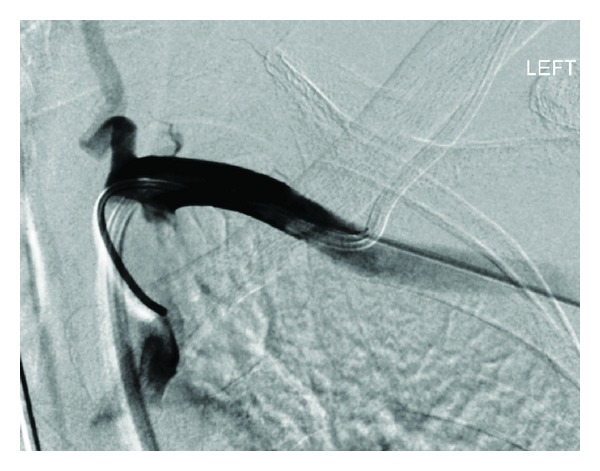
Catheter angiogram showing relation of catheter site to the left vertebral artery.

**Figure 7:**
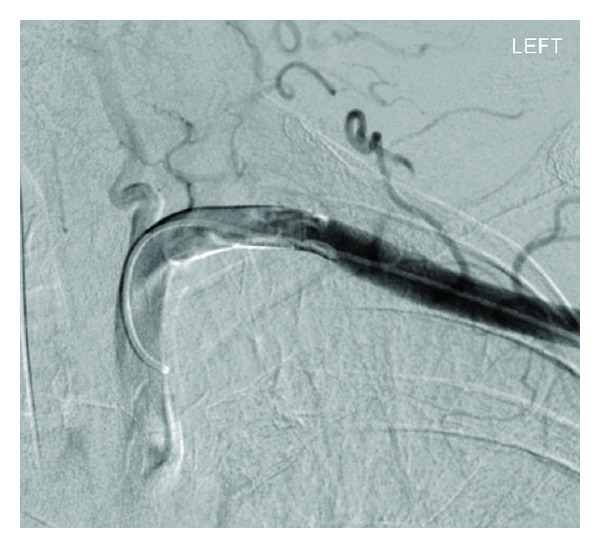
Catheter angiogram following deployment of Angio-Seal device.

Using ultrasound guidance, the left axillary artery was accessed, and an angiogram was performed with a 4 Fr angled catheter in the first part of the subclavian artery. This confirmed that the catheter had punctured the lateral segment of the subclavian artery, sufficiently distant to the vertebral artery origin. The subclavian catheter was then removed over a 50 cm (0.035 inch) Amplatz guidewire and an 8 Fr Angio-Seal device successfully deployed. Angiographic runs performed through the pigtail catheter confirmed successful closure of the puncture site (Figures 6 and 7), and pressure haemostasis was performed at the left axillary puncture site.

## 3. Discussion

The infraclavicular subclavian vein is one of the most frequently used sites for central venous cannulation [3]. This has a lower infection rate (4 per 100 catheter days) in comparison to the internal jugular vein (8.6 per 100 catheter days) [4] and is more readily accessible in trauma patients with cervical collars.

Accidental arterial puncture occurs in around 1% and 2.7% of jugular and subclavian approaches, respectively [5, 6]. This complication is often recognised early. However, in cases such as ours, with hypotensive and unstable patients, this can be more difficult to detect owing to the lack of pulsatile flow secondary to hypovolaemia.

There are a range of potential complications following inadvertent subclavian arterial puncture, including arterial occlusion, peripheral embolism, pseudoaneurysm formation, vessel laceration or dissection, and haemorrhage [7]. Some of these complications are limb or life threatening, particularly in critically ill patients or those with impaired coagulation.

When a line has been inadvertently inserted into an artery at a compressible site, this can be safely managed by removal and manual compression. However, if line removal is attempted at a noncompressible site, there is a greater propensity for serious complications such as haemorrhage or pseudoaneurysm to occur.

Inadvertent subclavian artery line insertion can be treated by either surgical or endovascular methods, or sometimes a combined approach is used. Surgical exposure of the intrathoracic segment necessitates a thoracotomy or sternotomy [8–10]. A supraclavicular incision may be required to access the distal postvertebral segment, with or without additional resection of the clavicle [9–11]. Patients requiring urgent central venous catheterisation are often critically ill with multiple comorbidities, hence surgical management represents a considerable undertaking.

There are a number of options in terms of endovascular management, including the use of stent grafts or percutaneous vascular closure devices. In the intrathoracic segment, stent grafting may be safely performed via ipsilateral axillary or femoral artery access [12]. The origin of the ipsilateral vertebral artery may need to be sacrificed to enable adequate placement of the stent graft. In elderly patients, patency of the contralateral vertebral artery should be ensured prior to stent graft deployment, although this is not always feasible in emergency situations.

The lateral aspects of the subclavian artery and vein are prone to compression between the clavicle and the first rib, particularly during arm abduction. Phipp et al. [13] have reported stent fracture in the lateral segment of the subclavian vessels. Given the frequency in which subclavian venous catheterisation is attempted at this site, stent grafting may not be the best option as first-line endovascular management for arterial cannulation.

Percutaneous closure devices have been used to close arterial punctures in both emergency and elective scenarios. The Angio-Seal device is designed to provide rapid and secure haemostasis after arterial puncture and is currently licensed for use in femoral arterial puncture sites, where it has been demonstrated to be both safe and effective [14, 15].

Angio-Seal devices were used in both of our cases, with removal of 7 Fr and 8 Fr catheters, respectively, and their use has been reported in the removal of lines up to 11.5 Fr [16]. There have also been previous reports of use of collagen-based [16–18] and suture-based devices [19, 20]. Angio-Seal has also been used in treatment of inadvertent subclavian arterial puncture during attempted internal jugular vein access [21].

We have reported two cases in acutely unwell and haemodynamically unstable patients in which Angio-Seal was used successfully. The lateral location of the arterial punctures in our cases precluded the intended use of covered stents in this location, given the risk of stent-related complications [13].

We conclude that a systematic approach is necessary for planning the treatment of inadvertent subclavian arterial cannulation. It is important to determine the exact location of the arterial puncture site and its relation to the ipsilateral vertebral artery, either with a preprocedural CTA or by means of angiographic runs performed at the beginning of the procedure. This would help plan the approach and also determine the nature of intended treatment. It is also vital to gain secure access across the puncture site that would enable deployment of a covered stent in the event of Angio-Seal failure.
